# Post‐Myocardial Infarction T‐tubules Form Enlarged Branched Structures With Dysregulation of Junctophilin‐2 and Bridging Integrator 1 (BIN‐1)

**DOI:** 10.1161/JAHA.116.004834

**Published:** 2017-05-04

**Authors:** Christian Pinali, Nadim Malik, J. Bernard Davenport, Laurence J. Allan, Lucy Murfitt, Mohammad M. Iqbal, Mark R. Boyett, Elizabeth J. Wright, Rachel Walker, Yu Zhang, Halina Dobryznski, Cathy M. Holt, Ashraf Kitmitto

**Affiliations:** ^1^ Division of Cardiovascular Sciences Faculty of Biology, Medicine and Health University of Manchester United Kingdom

**Keywords:** 3D electron microscopy, bridging integrator 1 protein, Eps 15 homology domain protein, heart failure, junctophilin‐2, myocardial infarction, transverse‐tubules, Heart Failure, Myocardial Infarction, Remodeling, Cell Biology/Structural Biology

## Abstract

**Background:**

Heart failure is a common secondary complication following a myocardial infarction (MI), characterized by impaired cardiac contraction and t‐tubule (t‐t) loss. However, post‐MI nano‐scale morphological changes to the remaining t‐ts are poorly understood.

**Method and Results:**

We utilized a porcine model of MI, using a nonlethal microembolization method to generate controlled microinfarcts. Using serial block face scanning electron microscopy, we report that post‐MI, after mild left‐ventricular dysfunction has developed, t‐ts are not only lost in the peri‐infarct region, but also the remnant t‐ts form enlarged, highly branched disordered structures, containing a dense intricate inner membrane. Biochemical and proteomics analyses showed that the calcium release channel, ryanodine receptor 2 (RyR2), abundance is unchanged, but junctophilin‐2 (JP2), important for maintaining t‐t trajectory, is depressed (−0.5×) in keeping with the t‐ts being disorganized. However, immunolabeling shows that populations of RyR2 and JP2 remain associated with the remodeled t‐ts. The bridging integrator 1 protein (BIN‐1), a regulator of tubulogensis, is upregulated (+5.4×), consistent with an overdeveloped internal membrane system, a feature not present in control t‐ts. Importantly, we have determined that t‐ts, in the remote region, are narrowed and also contain dense membrane folds (BIN‐1 is up‐regulated +3.4×), whereas the t‐ts have a radial organization comparable to control JP2 is upregulated +1.7×.

**Conclusions:**

This study reveals previously unidentified remodeling of the t‐t nano‐architecture in the post‐MI heart that extends to the remote region. Our findings highlight that targeting JP2 may be beneficial for preserving the orientation of the t‐ts, attenuating the development of hypocontractility post‐MI.

## Introduction

Heart failure is a multifactorial condition precipitated by a range of pathologies and is a secondary complication of myocardial infarction (MI).^1^ Loss of the transverse‐tubule (t‐t) network is an established feature of heart failure and is associated with impaired left ventricular (LV) contractility.^2^ T‐ts are specialized invaginations of the ventricular cardiac myocyte sarcolemma, extending into the interior of the cells to provide the infrastructure for conversion of the action potential to cardiac contraction through regulating Ca2^+^ influx.^3^ The architecture of the t‐t system varies between species, with a complex network of interconnected transverse and longitudinal invaginations (termed the transverse‐axial tubular system [TATS]) being a characteristic feature of small animals.^4^ In contrast, human cardiac t‐ts show no axial branching and adopt a radial pattern when the myocytes are viewed in cross‐section.^5^ Our previous study of the t‐t organization in sheep showed that the transverse invaginations have a spoke‐like morphology, as described for man; however, there was evidence of some longitudinal branching, although these elements accounted for less than 10% of the t‐t network.^6^

Confocal microscopy studies of a murine model of MI have revealed a reduction in t‐t density and patchy distribution in cardiac myocytes isolated from the peri‐infarct region, concomitant with dyssynchronous calcium (Ca^2+^) release and defective excitation‐contraction (E‐C) coupling.^7^ More‐detailed electrophysiological studies using a random access multiphoton microscope to measure membrane potentials at different sites simultaneously have further determined that as a consequence of structural remodeling, there is spatial and temporal impairment of the action potential propagation between the sarcolemma and elements of the TATS within cardiac myocytes from the peri‐infarct region of a rat model of ischemic heart failure.^8^, ^9^ Structural studies, using conventional transmission electron microscopy (TEM) to examine tissue sections taken from the peri‐infarct region from a rabbit model of MI, identified vacuoles that were suggested to be dilated remodeled t‐ts.^10^ However, the organization and architecture of these dilated t‐ts is not known, nor is it known whether these vacuoles correspond to collapsed t‐ts that are detached from the sarcolemma. Therefore, though t‐t loss post‐MI has been widely reported, there is limited information about the ultrastructural remodeling that occurs to the remaining t‐ts within the peri‐infarct and remote regions.

T‐t morphology and coordination are known to be regulated by a multitude of proteins.^11^ Notably, the scaffold protein, bridging integrator 1 (BIN‐1), has been shown to be central to t‐t biogenesis,^12^ trafficking of the L‐type voltage‐gated calcium channel to the t‐ts,^13^ and, more recently, folding of the t‐t inner membrane to limit ion diffusion.^14^ Depressed BIN‐1 expression has been identified in models of heart failure and linked to arrhythmogenesis.^15^, ^16^ The proteins, telethonin (T‐cap)^17^ and tropomyosin (Tpm),^18^ are proposed to link the t‐ts to the cytsoskeleton. Junctophilin‐2 (JP2), connecting the t‐ts to the junctional sarcoplasmic reticulum, is central for maintaining dyad organization (sites of Ca^2+^ release)^19^, ^20^ and organization of t‐t geometry.^21^ Reduced cardiac expression of JP2 has been identified both in animal models and patients with heart failure.^22^, ^23^, ^24^, ^25^, ^26^ A recent study of the t‐t network in murine skeletal muscle cells reported that the Eps 15 homology domain protein, isoform 1 (EHD1), is important for maintaining t‐t integrity through an association with BIN‐1. Depression of EHD1 expression in skeletal muscle myocytes resulted in upregulation of BIN‐1 and downregulation of JP2 with disordered and malformed t‐ts, leading to the proposal that EHD1 is a regulator of t‐t morphology.^27^ Whereas all 4 EHD protein isoforms (1–4) are present in the myocardium, the individual role of each isoform is yet to be delineated, although there is evidence suggesting a role in cardiac membrane protein targeting.^28^

Here, we have utilized a translationally relevant porcine model of MI, using an established nonlethal microembolization method to occlude the coronary artery to generate controlled microinfarcts.^29^, ^30^ Echocardiography showed that animals develop mild‐to‐moderate LV dysfunction, 1‐month post‐MI, recapitulating a commonly occurring condition in humans.^29^, ^30^ Using serial block face scanning electron microscopy (SBF‐SEM),^6^ we have investigated the t‐t nano‐architecture in healthy and post‐MI LV. We have further analyzed expression levels of proteins associated with tubulogenesis by combining biochemical and proteomic methods. Specifically, we sought to investigate the morphology of the t‐ts within (1) the peri‐infarct cardiac myocytes and (2) a region remote from the infarct epicenter, in order to determine whether cellular remodeling is spatially restricted, and (3) how changes to t‐t morphology are reflected by the expression profiles of proteins associated with t‐t structure and organization. Delineating t‐t ultrastructure within the LV post‐MI of a large animal model may provide new insights into how cellular remodeling contributes toward heart failure development in MI survivors.

## Methods

### Porcine Model of MI

Six pigs were used for this study (n=3 control, n=3 MI). All experiments were performed according to current UK Home Office regulations and under approval of the relevant University of Manchester (Manchester, UK) local ethics committee. After 24 hours of fasting, anesthesia was induced by inhalation (halothane 3–4%) only. Following intubation, anesthesia was maintained with a mixture of 1.5% halothane (Trofiels, Zug, Switzerland) and 98.5% oxygen (CFPO, Paris, France) with mechanical ventilation for the duration of the procedure. Perioperatively, end‐tidal CO_2_ levels (maintained between 26 and 36 mm Hg) and oxygen saturations (maintained at higher than 95%) were closely monitored. An electrocardiogram was also used to continuously monitor each animal's peri‐procedural cardiac status. Following coronary angiography, selective catheterization of left anterior descending branches was carried out for precise microembolization to induce mild‐to‐moderate LV dysfunction.^29^ Transthoracic echocardiography (TTE) was performed before and after each embolization procedure. At the end of the experimentation, all equipment was removed, hemostasis secured, and animals allowed to fully recover for the planned 4‐week duration. During this period, the overall health and well‐being of each animal was monitored daily for clinical signs of heart failure. At 4 weeks post‐MI and before euthanasia with a lethal intravenous administration of pentobarbital (18% solution), TTE was repeated. Hearts were removed and sampled as previously described.^29^ In brief, tissue was taken from 3 regions: the infarct, peri‐infarct (border), and remote regions. Samples taken from each section were divided for electron microscopy (EM), histological analysis, and with a portion snap frozen and stored at −80°C for protein analysis using western blotting or quantitative mass spectrometry (MS). For histological analysis, standard hematoxylin and eosin staining was used to define the infarct, peri‐infarct, and remote regions, after tissue fixation in formalin (10%), graded alcohol dehydration, xylene treatment, and paraffin embedding. Similarly, standard procedures were used for vimentin staining of tissue using a vimentin primary antibody (Santa‐Cruz 6260; Santa Cruz Biotechnology) and goat secondary (Texas Red conjugated) secondary antibody (Abcam 6787; Abcam, Cambridge, MA).^29^

### Three‐Dimensional Data Collection and Image Analysis

SBF‐SEM was used as previously described.^31^ Tissue blocks from the peri‐infarct, remote, and control myocardium were prepared and three‐dimensional (3D) data stacks were collected using an FEI Quanta 250 FEG SEM fitted with a Gatan 3View system. Serial images were collected at different magnifications, leading to voxel sizes ranging from 5.4 to 13.5 nm per pixel in the X‐Y plane with the cutting depth along the *Z*‐axis fixed at 50 nm. For feature measurements, the t‐t diameters and Z‐line spacing in control and MI (peri‐infarct and remote regions) LV were measured in multiple areas within a single cardiac myocyte and with multiple cells (3–5) per sample analyzed. For 3D reconstructions, the t‐ts were segmented and surface areas and volumes determined using IMOD^32^ and modeled using IMOD and Chimera.^33^ Tissue blocks from the peri‐infarct and remote regions for each animal and controls were sectioned, and 100‐nm slices were examined using a TEM FEI Tecani 12 (×13 500) to investigate the t‐t lumen.

### Western Blotting

Western blotting was conducted as previously described using a total protein loading method^34^ and an internal standard to normalize loading between gels,^21^ because the proteins typically used as loading controls (eg, GAPDH) were found to be modulated in the post‐MI heart.

### Quantitative Mass Spectrometry and Proteomic Analysis

Fifteen milligrams of pig tissue from control, peri‐infarct, and remote regions were lysed in 500 μL of ice‐cold 25 mmol/L of ammonium bicarbonate supplemented with a protease inhibitor cocktail (Roche, Indianapolis, IN). Rapigest (for 0.05%) was added, samples were heated to 80°C for 10 minutes, then reduced and alkylated with 3 mmol/L of DTT and 9 mmol/L of iodoacetamide, respectively. Fifty micrograms of protein was digested in 25 mmol/L of ammonium bicarbonate overnight at 37° with trypsin. Digested samples were analyzed by liquid chromatography with tandem MS (LC‐MS/MS) using an UltiMate 3000 Rapid Separation LC (RSLC; Dionex Corporation, Sunnyvale, CA) coupled to an Orbitrap Elite (Thermo Fisher Scientific, Waltham, MA) mass spectrometer. The acquired MS data were analyzed using Progenesis QI for proteomics (v2.0; Nonlinear Dynamics, Newcastle Upon Tyne, UK). Retention times in each sample were aligned using one LC‐MS run as a reference, then the “Automatic Alignment” algorithim was used to create maximal overlay of the two‐dimensional (2D) feature maps. The resulting peaklists were searched against the Uniprot Pig database (version 2013‐5) using Mascot v2.4.1 and the results imported into Progenesis LC‐MS for annotation of peptide peaks. The identification of 3 unique peptides was used as an inclusion criterion and *P*<0.05 (ANOVA) as a measure of significance to changes in protein abundance.

### Immunofluorescence Labeling

Tissue sections from the peri‐infarct region and comparable anatomical area within the control LV were immunolabled using previously established methods.^35^ Fixed tissue sections were incubated with the primary antibodies (anti‐JP2, anti‐RyR2 [ryanodine receptor 2], and anti‐EHD2) overnight at 4°C, using a dilution of 1:100. Secondary IgGs (Alexa Fluor 488 anti‐Goat [JP2], Cy3 anti‐Rabbit [EHD2], and FITC anti‐Mouse [RyR2]) were added for 1 hour. Labeled sections were then imaged on a Zeiss LSM5 PASCAL (Carl Zeiss Microscopy, Jena, Germany) laser scanning confocal microscope. No labeling above background was obtained when the primary or secondary antibodies were omitted (data not shown).

### Statistical Analysis

Mean value±SEM was calculated for each morphological feature from each control pig (n=3). A one‐way ANOVA showed no interanimal differences for Z‐line spacing or t‐t diameter measurements in each of the control animals (*P* values, 0.2541 and 0.0848, respectively); therefore, the mean from each control animal was combined and averaged to generate an overall mean value. An unpaired Student *t* test was used to compare the mean t‐t diameter from control pigs (n=3) with our previously published sheep t‐t diameters.^6^ Mean values for t‐t diameter and Z‐line spacing within the peri‐infarct and remote regions of the MI pigs (n=3) were initially calculated for each animal as described for control, with no significant interanimal variation determined leading to the calculation of an overall mean value±SEM (n=3) as reported in the Results section. Unpaired Student *t* tests of the mean values from each pig comparing control with peri‐infarct and control with remote regions were used to test changes to the morphological features post‐MI. We additionally used two paired Student *t* tests to investigate whether t‐t measurements were statistically different between the peri‐infarct and remote regions within the same MI pig. All data were analyzed using GraphPad Prism software (version 7.0; GraphPad Software Inc., La Jolla, CA).

## Results

### T‐t Organization in the Healthy Pig Heart Mirrors That Reported in Human Hearts

**Figure 1:**
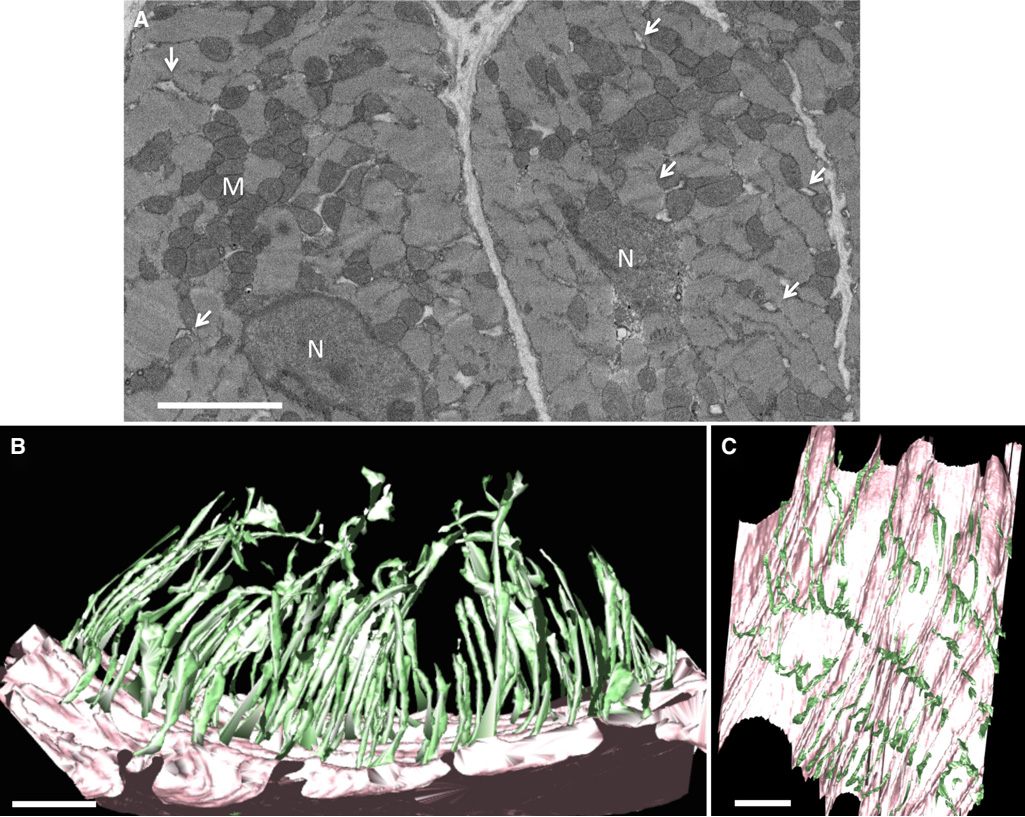
Pig t‐ts form a radial distribution in control cardiac myocytes A, An exemplar portion of a serial SEM image from a volumetric stack illustrating the details of the cardiac myocyte ultrastructure. M, mitochondria; N, nucleus. Arrows indicate t‐ts. Scale bar=5 μm. B, Segmentation of the sarcolemma (pink) and t‐tubules (green) illustrating a radial organization extending from the exterior to interior of the cell. C, View of the t‐t network orthogonal to the sarcolemmal plane illustrating the regular spacing and organization of the t‐ts. Scale bar=2 μm. SEM indicates scanning electron microscopy.

The 3D structure of healthy pig t‐ts in tissue sampled from the LV (apex; Figure 1) revealed that the t‐ts adopt a radial configuration consistent with that reported for man^5^ and as we have previously described for sheep.^6^ The pig t‐t mean diameter, 188±17 nm (133 tubules measured; n=3 pigs), is similar to our previous reports for sheep cardiac t‐ts, 244±64 nm (*P*>0.05). Other structural features common to the sheep t‐t organization are (1) t‐ts are not connected to adjacent tubules, (2) each t‐t is punctuated by regional points of dilation (nodules), and (3) the presence of “twin” t‐tubules, where two invaginations extend from the sarcolemma at the same position at the Z‐line. However, in contrast to sheep t‐t nano‐architecture, the pig t‐t network has no longitudinal branches.^36^, ^37^

### T‐ts Within the Peri‐Infarct Cardiac Myocytes Form Enlarged, Highly Branched Structures

**Figure 2:**
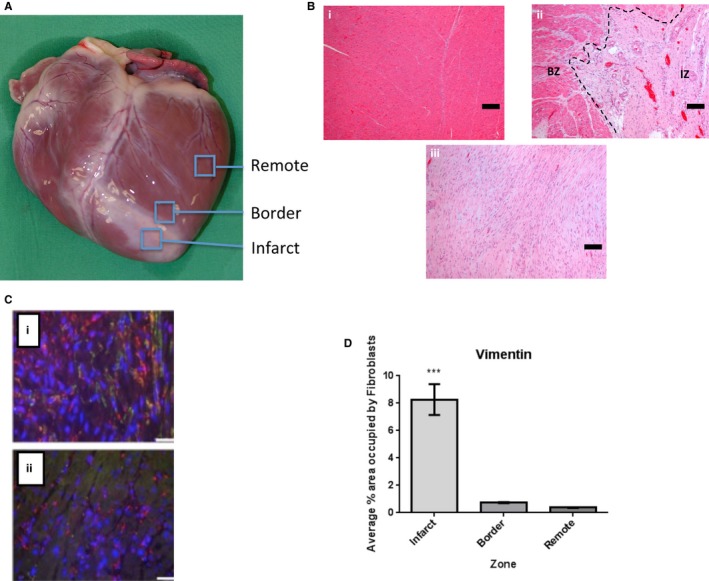
Tissue‐level characterization of the porcine heart post‐MI. A, Image of an explanted pig heart 4 weeks post‐MI showing an area of infarct distinguished by a pale region of ischemic tissue; the peri‐infarct region (also termed border zone) directly adjacent to the infarct and remote region of the myocardium. B, Histological analysis of pig myocardium 4 weeks following infarction. (i) Remote area of myocardium showing normal cardiac myocytes. (ii) Section showing border zone (BZ) adjacent to infarct zone (IZ) with the dashed line illustrating a clear demarcation between the zones. The infarct zone is characterized by pale stained tissue containing inflammatory cells and neovessels. (iii) Infarct zone showing pale stained necrotic tissue with some inflammatory cell infiltration. Scale bar=100 μm. C, Fibroblast recruitment post‐MI in the pig. Fibroblasts immunopositive for vimentin stained red and alpha‐actin (green). Example immunostaining in the infarct zone 4 weeks postinfarct in the infarct zone (i) and corresponding border zone (ii). Scale bar=25 μm. D, Quantification of the myofibroblasts reveals a greater proportion within the infarct region compared to border and remote zones (***P<0.001). MI indicates myocardial infarction.

**Figure 3:**
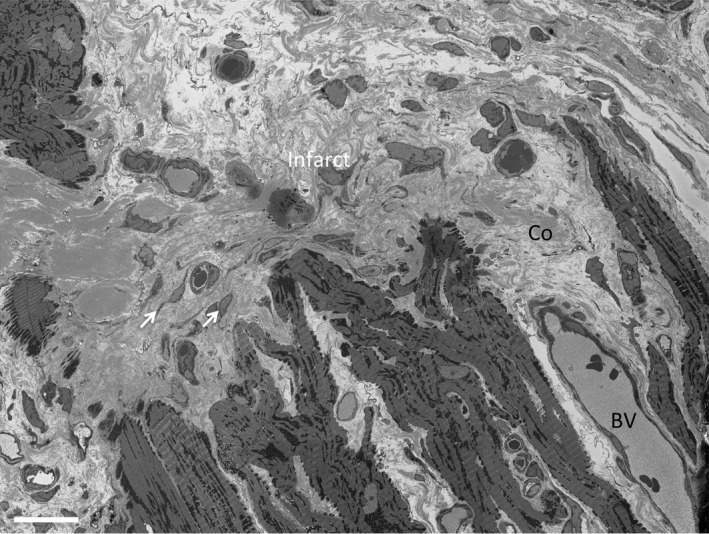
Infarct region is devoid of cardiac myocytes. An exemplar portion of a serial SEM image from a volumetric stack illustrating a portion of the infarct region and the surrounding peri‐infarct cardiac myocytes. The infarct is composed primarily of collagen and fibroblasts. Scale bar=20 μm. BV indicates blood vessel; Co, collagen; SEM, scanning electron microscopy.

**Figure 4:**
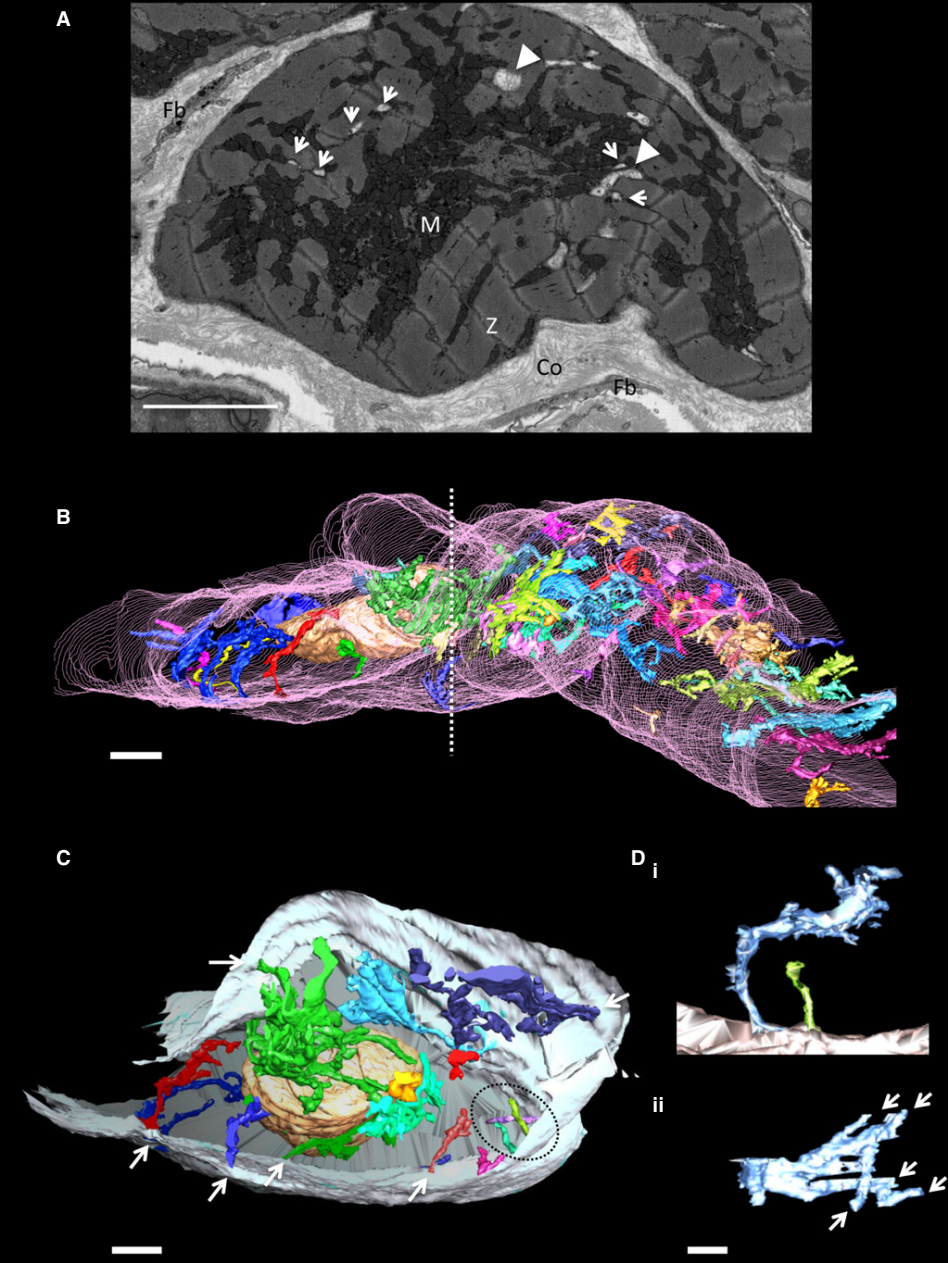
Highly branched enlarged t‐tubules form within the peri‐infarct cardiac myocytes. A, Exemplar serial SEM image showing a cardiac myocyte bordering the infarct. Arrows highlight t‐ts with dimensions typically found in control, whereas solid arrow heads indicate what appear in 2D to be vacuoles. 3D reconstruction shows that these “vacuoles” are part of enlarged highly branched remodeled t‐ts. M, mitochondria; Z, Z‐line; Co, collagen; Fb, fibroblast process. Scale bar=5 μm. B, 3D reconstruction of the t‐ts within peri‐infarct cardiac myocytes. The t‐ts form “overgrown” structures; each separate “overgrown” t‐t is segmented in a different color; the sarcolemma is segmented in pink. Scale bar=5 μm. The dashed line indicates the position of the cross‐section through the cell shown in (C). C, In this view, the remodeled t‐ts can be seen to be attached to the sarcolemma (gray) as indicated by the arrows. There are regions where there are no t‐ts and the spacing of the remodeled t‐ts is irregular. The t‐ts enclosed by the dashed ellipsoid indicate t‐ts with a morphology comparable to control t‐ts. Scale bar=2 μm. D, (i) Shows a remodeled t‐t (blue) adjacent to a t‐t (green) with dimensions similar to control. (ii) Top view of the enlarged, branched invagination revealing how it is formed from multiple, smaller, t‐ts. Scale bar=500 nm. 2D indicates two‐dimensional; 3D, three‐dimensional; SEM, scanning electron microscopy.

Examination of the explanted experimental hearts allowed visual identification of the regions of ischemic damaged tissue as indicated in Figure 2A. Histological analysis of the infarct, the peri‐infarct and remote regions showed a cellular composition consistent with previous assignments, that is, the infarct is highly populated by fibroblasts and collagen deposition as indicated by elevated levels of the fibroblast marker vimentin (Figure 2B through 2D). Figure 3 shows an exemplar serial SEM image of the peri‐infarct zone adjacent to the scar with the image in Figure 4A showing, at higher magnifications, a representative cardiac myocyte within this region. Measurement of the Z‐line spacing within the peri‐infarct region cardiac myocytes revealed that the sarcomere lengths were comparable to that of control; 1936±10 nm (total of 574 sarcomere lengths measured; n=3) peri‐infarct and 1831±24 nm (total of 216 sarcomere lengths measured; n=3) control. However, when we segmented the t‐ts, 3D reconstructions revealed regions of the cells without tubules in agreement with previous findings from studies of other species, mainly small animal models of MI.^7^ Therefore, our data now show that t‐t loss post‐MI is also a feature of the porcine heart, and, importantly, we report, for the first time, that the majority of the remaining t‐ts are remodeled to form disordered, enlarged structures (Figure 4B) that remain attached to the sarcolemma (Figure 4C). Closer examination of the “overgrown” t‐t structures found that they are formed by a series of interconnected smaller tubules, forming a complex branched morphology (Figure 4D and Video S1). These smaller “fused” t‐ts, with a mean diameter of 178±13 nm (total of 72 tubules measured; n=3), are similar to control t‐ts (*P*>0.05), but the junctions, where these branched t‐ts coalesce, have larger diameters of 1018±62 nm (n=21; *P*<0.0001).

### T‐ts Within the Remote Region Are Remodeled

**Figure 5:**
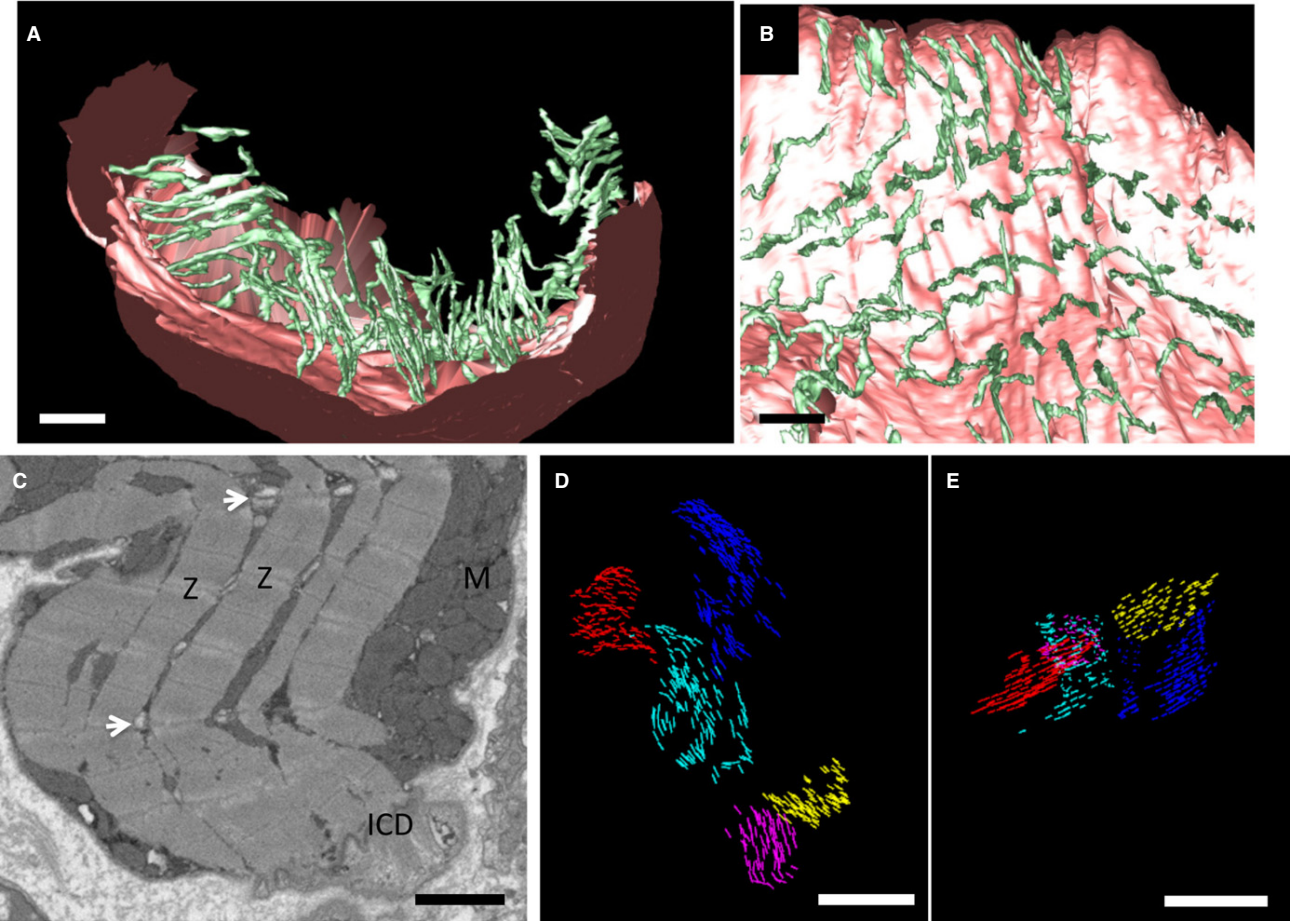
3D reconstruction of the t‐t morphology and arrangement in the remote region. A, The t‐ts (green) adopt a radial configuration extending from the sarcolemma (pink) toward the center of the cell, stopping where there is a nucleus. B, View of the t‐t organization orthogonal to the sarcolemma illustrating the regular spacing of the invaginations. Scale bar=2 μm. C through E, Morphometric analysis of Z‐line spacing. C, Portion of a serial SEM image of the remote region illustrating how the sarcomere features are well defined with the Z‐lines identifiable as a dark band with a lighter I‐band flanking on either side. The central M‐band is also resolved. ICD; intercalated disc: M; mitochondria. D and E, Shows 2 views of how the sarcomere spacing was measured using IMOD, regions of 5 separate cells (indicated by the different color) were analyzed. Z‐lines were measured at 1‐μm intervals in the Z‐direction. Scale bar=2 μm. 3D indicates three‐dimensional; SEM, scanning electron microscopy.

3D segmentation of SBF‐SEM volumetric data revealed that the t‐ts in the remote region have a similar radial orientation as in control tissue (Figure 5A) and are regularly spaced (Figure 5B). Similarly, the Z‐line spacing was consistent with sarcomere morphology in control (1822±55 nm, total of 905 sarcomere lengths measured; n=3); see Figure 5C through 5E for exemplar images showing Z‐line sampling. However, the remote region t‐ts are narrower (136±7 nm, total of 75 tubules measured; n=3) compared to control (*P*=0.0063), indicating that remodeling has occurred, although the volume and surface areas are unchanged (Table). A paired Student *t* test was used to investigate whether there may be differences between the t‐t diameters in the peri‐infarct and remote regions within the same pig. The analysis showed narrowing of the t‐ts within the remote region as a consistent feature of the MI pig (*P*=0.0439).

**Table 1:** T‐Tubule Properties Determined From SBF‐SEM and TEM Analyses

Tissue	No. of t‐ts	Mean Surface Area (nm^2^)	Mean Volume (nm^3^)
Control	103	2.54×10^6^±0.13×10^6^	6.02×10^7^±3.68×10^6^
Peri‐infarct	63	3.40×10^7^±7.28×10^6^ (a)	7.63×10^9^±4.82×10^7^ (b)
Remote	135	4.01×10^6^±0.23×10^6^	1.72×10^8^±1.06×10^7^

N=3 pigs. All data compared to control and expressed as mean±SEM. SBF‐SEM indicates serial block face scanning electron microscopy; TEM, transmission electron microscopy.

a *P*<0.05.

b *P*<0.001.

### BIN‐1 and JP2 Protein Expression Dysregulation Within the Peri‐Infarct and Remote Regions

**Figure 6:**
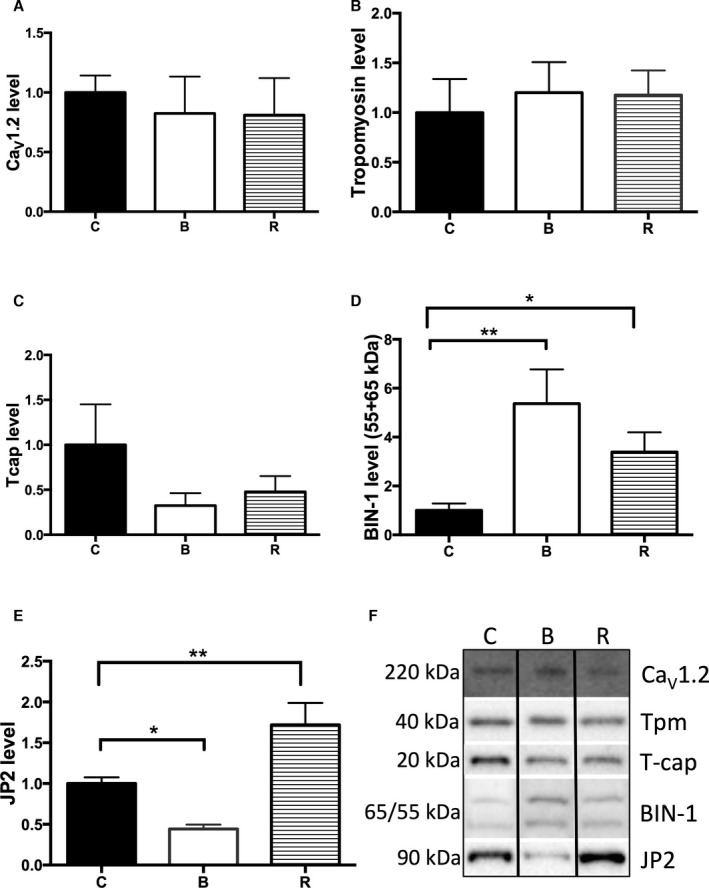
Protein profiling of the tissue lysate from control pigs compared to the peri‐infarct and remote regions post‐MI using western blotting techniques. The expression levels of the following proteins were analyzed: A, CaV1.2; ion channel subunit of the L‐type voltage‐gated calcium channel B, Tropomyosin C, Tcap; telethonin D, BIN‐1; Bridging integrator 1 E, JP2; junctophilin‐2 F, Exemplar western blots for each of the proteins analyzed. There is an imbalance between JP2 and BIN‐1 expression levels within the peri‐infarct and remote regions. Separate analysis of the BIN‐1 55‐ and 65‐kDa bands showed the same change as when combined. B; border zone (peri‐infarct region), R; remote region, C; control. (*P<0.05, **P<0.01)

Using quantitative MS, we identified several proteins associated with E‐C coupling localized to the dyadic cleft. Comparison with the control protein expression profiles revealed no change to the abundance of the RyR, the Ca^2+^ATPase pump (SERCA2a), and Ca^2+^ calmodulin‐dependent protein kinase II (CaMKIIδ) in either the peri‐infarct or remote regions. The auxiliary extracellular subunit, α1δ2, but not Ca_v_1.2 (calcium channel, voltage‐dependent, L type, alpha 1C subunit), of the L‐type voltage‐gated calcium channel (localized to the sarcolemma and t‐ts) was identified in the peri‐infarct and control samples, but showed no change in abundance. Given the 1:1 stoichiometry between Ca_V_1.2 and α1δ2, we inferred that there was no change to Ca_V_1.2 expression and confirmed this assumption by western blotting (Figure 6A). The cytoskeletal proteins, Tcap and tropomyosin, were detected by MS in the remote and border regions, but less than 3 unique peptides were identified with *P*>0.05 and thus no conclusions could be drawn. However, western blotting showed no change to tropomyosin expression (Figure 6B) and identified a downward trend in Tcap levels within both the peri‐infarct and remote zones (Figure 6C; *P*=0.06). BIN‐1 was not detected by MS, but western blotting (Figure 6D) revealed an upregulation in both the peri‐infarct and remote regions, +5.4 (*P*<0.01) and +3.4 (*P*<0.05), respectively. Within the remote region, the MS data indicated JP2 was upregulated +1.7‐fold, a result confirmed by western blotting (Figure 6E; *P*<0.01) and additionally revealed an ≈ −0.5‐fold reduction within the peri‐infarct area (Figure 6E) (*P*<0.001) compared to control tissue. Proteomics identified EHD isoforms 1, 2, and 4 in all sample groups, revealing that only EHD2 levels changed by −1.7‐fold (*P*=0.04) within the peri‐infarct region. In the remote region, EHD1, 2, and 4 were unchanged, although EHD4 trended down (−1.8‐fold; *P*=0.053). Less than 3 peptides for EHD3 were identified in each sample, so no conclusions as to expression‐level changes could be reliably drawn.

**Figure 7:**
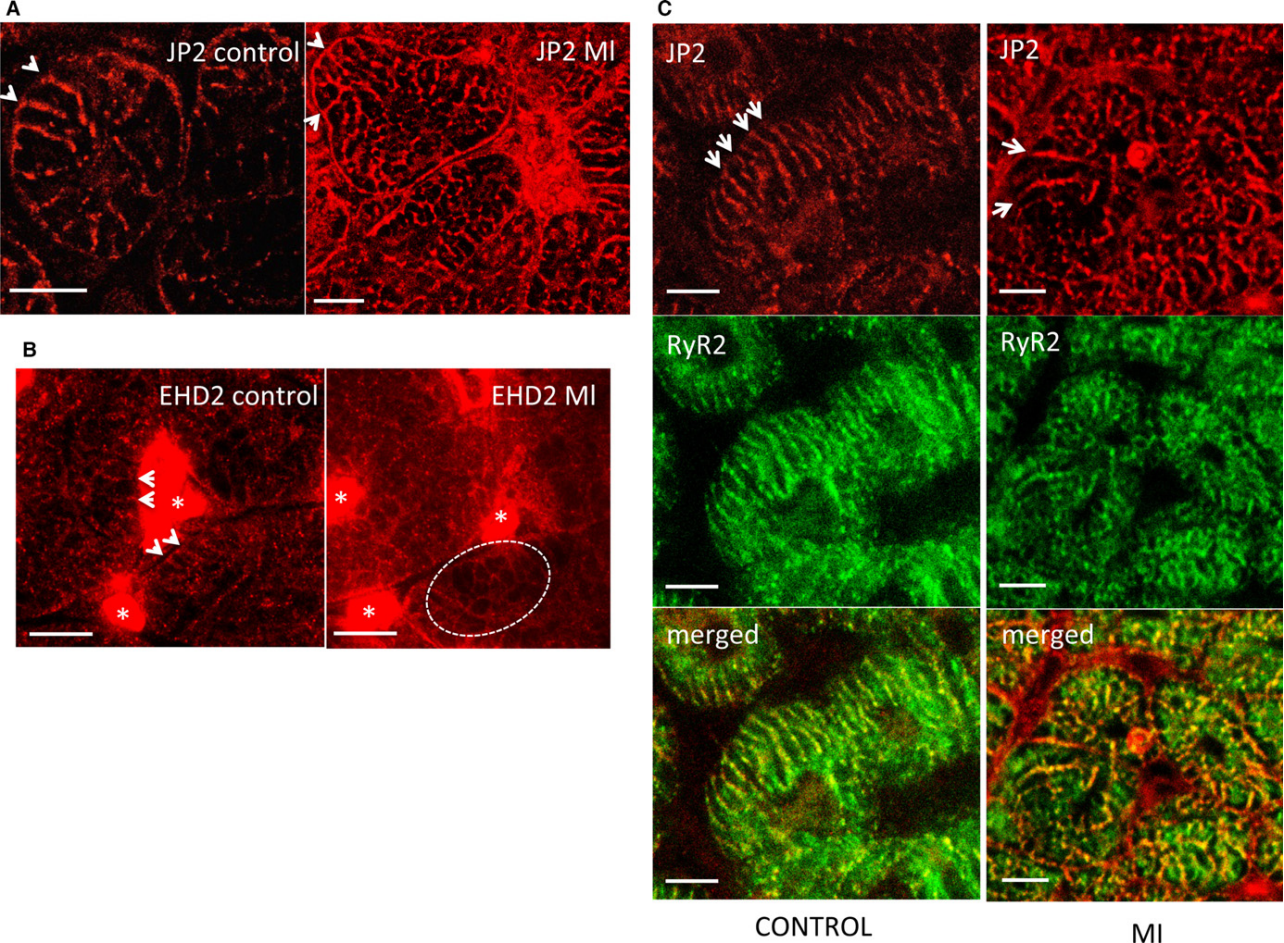
Immunolabeling of JP2, EHD2, and RyR2 in tissue sections from control and MI LV (peri‐infarct region). A, Representative confocal image of a tissue section with immunolabeling of JP2. JP2 is distributed in the control LV along each t‐t trajectory, the t‐t network has a radial organization in the transverse orientation in agreement with the higher‐resolution SBF‐SEM analyses. The right panel (peri‐infarct tissue) in agreement with the EM data now shows JP2 labeling along enlarged, branched irregular structures. B, EHD2 is accumulated between cells (highlighted with an asterisk [“*”]) with only weak staining within cardiac myocytes. The staining pattern in places is consistent with putative t‐ts (indicated by arrows). In the peri‐infarct region, the EHD2 labeling shows a complex organization. Dashed ellipsoid highlights putative labeling of remodeled t‐ts. C, Dual labeling of JP2 and RyR2 shows colocalization along the structures typical of t‐ts. In the peri‐infarct region, colocalization is maintained along the remodeled t‐ts. EHD2 indicates Eps 15 homology domain protein, isoform 2; EM, electron microscopy; JP2, junctophilin‐2; LV, left ventricle; MI, myocardial infarction; RyR2, ryanodine receptor 2; SBF‐SEM, serial block face scanning electron microscopy.

Based upon these data, we next undertook immunolabeling and confocal microscopy to interrogate RyR2, JP2, BIN‐1, and EHD2 distribution within the peri‐infarct region. Whereas primary antibodies for RyR2, JP2, and EHD2 gave strong fluorescence, we did not find a suitable commercial antibody for BIN‐1. Significantly, we found that the remaining population of JP2 was coincident with the morphology of the “overgrown” branched structures identified by EM, which would suggest that there may be jSR (dyads) formed along these tubules (antibody Santa Cruz, sc‐51313 [Santa Cruz Biotechnology], raised against the C‐terminus of JP2; Figure 7A). In control tissue, EHD2 was most abundant between cardiac myocytes with comparatively weak staining of the interior of the cell (Figure 7B). However, close examination of the cellular staining did find a labeling pattern in keeping with t‐t‐like invaginations, although the distribution appeared more complex, becoming more intricate in the peri‐infarct cardiac myocytes. Because of the complex distribution of EHD2, the data are equivocal as to whether EHD2 is a t‐t protein and/or is localized to other intracellular structures. Dual labeling of JP2 with RyR2 within control cardiac tissue showed colocalization along the t‐ts. Furthermore, colocalization was also evident within the enlarged t‐ts within the peri‐infarct region (Figure 7C), indicating that RyR2 is still closely associated with the remodeled t‐ts.

### T‐ts in the Peri‐Infarct and Remote Regions Have a Densely Folded Inner Membrane

**Figure 8:**
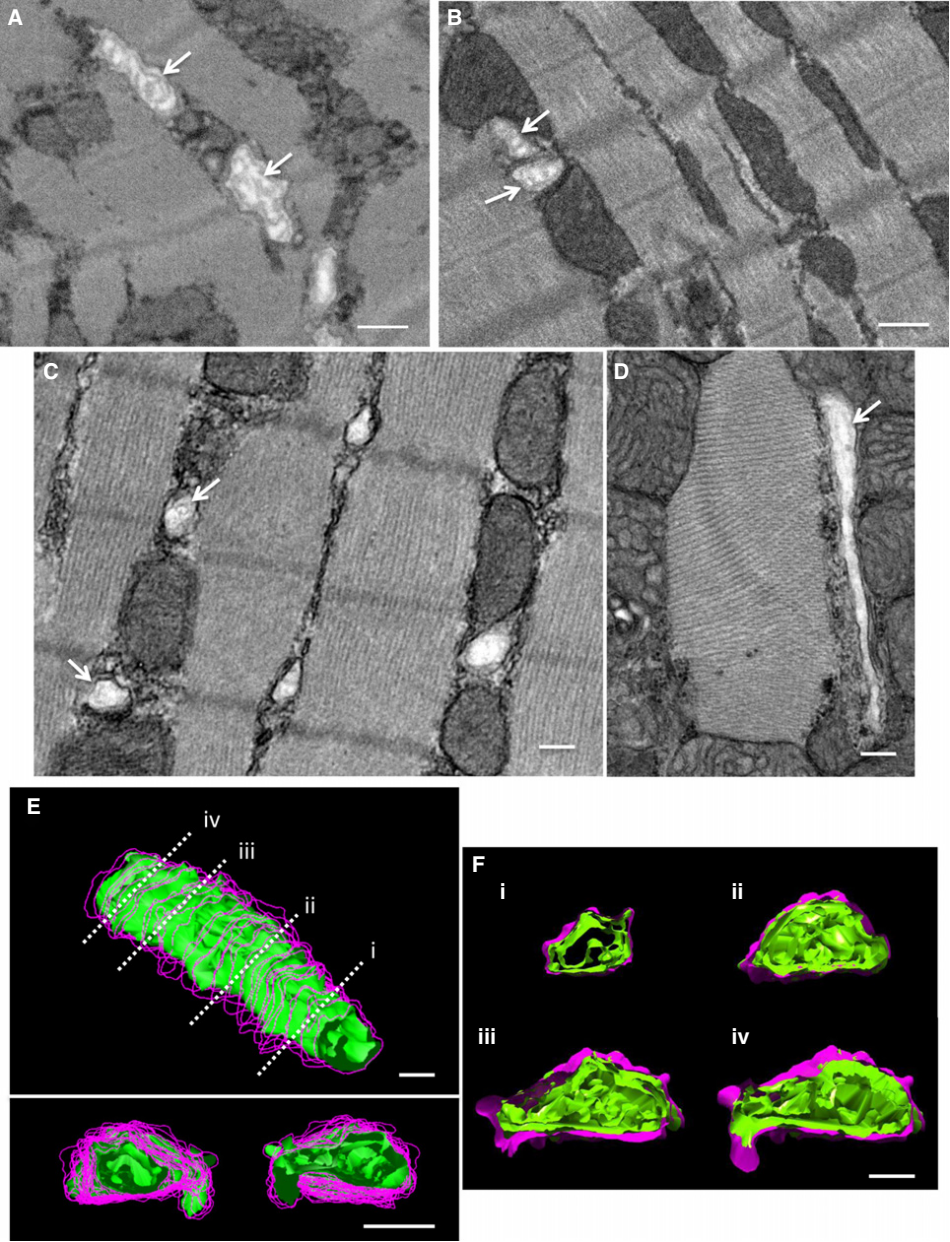
T‐ts have an intricate folded inner membrane. A, Exemplar serial SEM image of remodeled t‐ts within the peri‐infarct region. Arrows highlight the folds within the t‐ts. B, Exemplar serial SEM image of a “twin t‐t” within the remote region post‐MI. Arrows highlight the folds within both t‐ts. C, TEM image of control tissue showing several t‐ts in cross‐section. The inner membrane folds are clear in some t‐ts (indicated by arrows). D, TEM image of a control t‐t (partial transverse view) showing that, in some regions, the basal lamina is well defined, but in other parts, although not empty, the interior appears “fuzzy.” E, 3D reconstruction of a portion of a remodeled t‐t within the peri‐infarct region. The outer membrane envelope is shown in purple contours, and the inner folds are shown as green isosurface. The t‐t as viewed from both ends are shown in the panel below. F, (i–iv) correspond to cross‐sections through the t‐t as indicated by the dashed lines in (E). The inner membrane is formed by a high density of folds. Scale bars=200 nm. 3D indicates three‐dimensional; MI, myocardial infarction; SEM, scanning electron microscopy; TEM, transmission electron microscopy; t‐ts, transverse‐tubules.

SBF‐SEM data sets collected at higher magnifications (5.5–6.5 nm/pixel in the X‐Y plane) revealed the interior of the t‐ts, showing that the peri‐infarct “overgrown” t‐ts and remote region t‐ts contained a dense inner membrane network (Figure 8A and 8B). There was no evidence of similar luminal structures within the control t‐ts. To rule out differences in resolution and stain contrast as a reason for why the inner membrane fold was absent in control t‐ts samples, we examined thin sections by higher‐resolution TEM. As can be seen in Figure 8C and 8D, the basal lamina is resolved in control t‐ts, but the interior is characterized by a “fuzzy” content lacking definition, a morphology consistent with previous reports.^14^ Therefore, these data suggest that the internal structures observed within the peri‐infarct and remote region cardiac myocytes are a development post‐MI. Segmentation of the luminal densities within the remodeled peri‐infarct t‐ts revealed dense fold‐like structures (Figure 8E and 8F).

## Discussion

This study has generated the first highly detailed 3D structures for the t‐t network within the porcine LV, showing that they adopt a radial configuration, a morphology described in the human heart. We found no evidence of branching in the pig cardiac myocytes, a feature we identified using the same technique in the sheep.^6^ However, we did identify “twin” tubules, a feature common to both large animals, structures that will lead to an increased density of invaginations between the Z‐lines, and thus, we propose, may provide additional routes for electrical propagation within the myocyte when there is little or no longitudinal branching. Importantly, we now report, and characterize, that in addition to t‐ts loss within the peri‐infarct cardiac myocytes of a porcine MI model, the remnant t‐ts are intricately remodeled, with both structural and molecular changes. A feature of the remodeled t‐ts is a highly complex branched structure. Interestingly, studies of small animal models of heart failure have reported a loss of transverse tubules with an increase in the number of longitudinal elements.^38^ This might suggest that despite interspecies differences between t‐t architecture, a common pathological response is the formation of branched axial structures.

Additionally, we show that t‐t remodeling also extends in the remote region, with narrowing of the invaginations when compared to control. Dilation of t‐ts has been reported as a consequence of pathological remodeling in animal models of heart failure^39^ and patients with ischemic and dilated cardiomyopathy.^40^ We have previously reported that cardiac myocytes within the LV of a tachypacing sheep model of end‐stage heart failure are characterized by regions where t‐ts are narrowed at the sarcolemma, but form enlarged dilated sac‐like structures toward the interior of the cell.^6^ Thus, narrowed t‐ts in the remote region in the post‐MI porcine heart with mild‐to‐moderate LV dysfunction may reflect the first stages of t‐t remodeling. Therefore, the data presented here suggest that strategies aimed at t‐t regeneration in the post‐MI heart needs to also take into account the presence of the remaining remodeled t‐ts and the potential contribution to aberrant Ca^2+^ movements. This conclusion is reinforced by the work of Sacconi et al,^8^, ^9^ who have demonstrated that, within isolated cardiac myocytes from a rat MI model, there are electrical uncoupled transverse‐axial tubules resulting in failure of the action potential propagation. Additionally, these investigators show that there are regions of spontaneous electrical activity at sites along t‐ts, with amplification of Ca^2+^ sparks resulting in asynchronous, proarrhythmogenic Ca^2+^ waves. These pathoelectrophysiological events are suggested to be a consequence of structural remodeling underlying contractile dysfunction.

### Changes to the “t‐tubulome” Protein Composition Concomitant With t‐t Remodeling

BIN‐1 expression has previously been reported to influence folding of the inner t‐t inner membrane, with ablation leading to loss of the luminal lining.^14^ Although we do not identify an extensive inner fold structure within control t‐ts (also absent in healthy sheep t‐ts^6^), our new data reporting that BIN‐1 is upregulated within the post‐MI LV is consistent with an overdeveloped internal membrane system within the t‐t lumen. These dense folds may lead to restricted diffusion of molecules into the t‐ts and thus contribute toward dysfunctional properties in both the peri‐infarct and remote regions. Whereas a loss of JP2 within the peri‐infarct cardiac myocytes will impair dyad formation, immunofluorescence labeling shows that there still remains populations of JP2 and RyR2 associated with the remodeled t‐ts, consistent with electrophysiological studies showing that there are regions of aberrant Ca^2+^ release,^8^, ^9^ with the t‐ts also attached to the sarcolemma. Therefore, in conclusion, we show here a previously unrecognized complex remodeling process, with both t‐t loss and structural and molecular changes to the remnant t‐ts occurring, features that will compound the development of dyssynchronous and aberrant Ca^2+^ dynamics previously described in the peri‐infarct region post‐MI.^7^, ^8^, ^9^, ^41^

Skeletal muscle from *Ehd1‐*heterozygous–deficient mice has been shown to express high levels of BIN‐1 (+2.25‐fold) with a large depression (−13‐fold) of JP2 compared to controls. In these mice, the t‐t system was disorganized and characterized by what the investigators termed overgrowth and aggregation, although they did not report any 3D structural data.^27^ Whereas the porcine MI heart shows no change to EHD1 abundance, EHD2 levels are depressed with upregulation of BIN‐1 and reduction in JP2, and therefore future studies investigating t‐t ultrastructure may benefit from also focusing upon the role of EHD2.

The overgrown t‐ts in the peri‐infarct region, though still attached to the sarcolemma, are no longer spatially confined, and we propose that, in part, disorder is consistent with a depletion of JP2 levels and thus loss of the anchor stabilizing the t‐t trajectory.^21^ Transgenic mice with cardiac‐specific JP2 upregulation were shown to feature a persevered, well‐aligned t‐t architecture when subjected to LV pressure overload, compared to control animals, leading to the proposal that JP2 is beneficial for attenuating heart failure development.^42^ Consistent with JP2 having a cardioprotective role by preserving t‐t orientation, we have found that within the remote region where the JP2 levels are increased (+1.7‐fold), the radial configuration of the t‐ts is maintained, despite elevated BIN‐1 levels. Significantly, a recent study has shown that adeno‐associated virus‐mediated overexpression of JP2 (2.38±0.61‐fold increase in abundance) in mice with early‐stage heart failure leads to a suppression of Ca^2+^ leak through the RyR2 and a persevered t‐t organization compared to control groups.^43^ Therefore, our data now provide additional evidence to indicate that there may also be benefits for targeting JP2 expression in the setting of MI.

In summary, the data here provide new insights into t‐t remodeling at both the structural and molecular level in the infarcted porcine heart and, importantly, characterize t‐t dysmorphology. Differential protein expression levels through the LV suggest that longer term studies may be of value to determine whether, at more‐advanced time points, further changes to the BIN‐1‐JP2 protein axis leads to a critical tipping point that precipitates a more‐severe and more‐widespread t‐t structural phenotype, providing the impetus for hypocontractility and heart failure development.

## Sources of Funding

This work was supported in part by the British Heart Foundation RG/11/2/28701 (Kitmitto).

## Disclosures

None.

## Supporting information


**Video S1.** Examples of enlarged, disordered, t‐ts within the peri‐infarct cardiac myocytes, and how they are composed from multiple smaller tubules to form complex branched structures. Sarcolemma not shown for clarity.
